# Extracellular bioelectrical lexicon: detecting rhythmic patterns within dermal fibroblast populations

**DOI:** 10.1038/s41598-025-15071-z

**Published:** 2025-08-14

**Authors:** Rute C. Félix, Maria C. Medeiros, Youssef Elamine, Deborah M. Power, Henrique Leonel Gomes

**Affiliations:** 1 Centro de Ciências do Mar (CCMAR/CIMAR), Universidade do Algarve, Campus de Gambelas, Faro, 8005-139 Portugal; 2 Faculdade de Ciências e Tecnologia, Universidade do Algarve, Faro, 8005-139 Portugal; 3 Departamento de Engenharia Electrotécnica e de Computadores, Instituto de Telecomunicações, Coimbra, 3030-290 Portugal

**Keywords:** Electrophysiology, Non-excitable cells, Extra-cellular electrodes, Bioelectricity, Fibroblasts, Biophysics, Systems biology, Electrical and electronic engineering

## Abstract

This study uses a bioelectronic-based method to establish how non-electrogenic cells, like dermal fibroblast, employ bioelectrical signals to convey information. Electrophysiology using large-area Multielectrode Arrays (MEAs) devices revealed how populations of non-electrogenic cells in vitro generate patterns of bioelectrical signals. The period of the bioelectrical patterns depends on cell population activity. In a fully formed, healthy monolayer, bioelectrical activity is minimal. But during the formation of a monolayer, signals appear randomly, with a dominant period of 4.2 min. Occasionally, quasi-periodic bursts occur with a period between 1.6 and 2 min. When a mechanical wound is inflicted and during subsequent monolayer repair, quasi-periodic signal bursts occur, with an average period ranging from 60 to 110 min. The study uncovers a short-range non humoral communication system and a lexicon of bioelectrical signals linked to cell states.

## Introduction

Understanding the mechanism by which cell populations coordinate their activities to accomplish multicellular tasks remains an unsolved question in Biology. Recent studies have put forward the hypothesis that, akin to nerve and neural cells, non-excitable cells also utilize bioelectricity to transmit information across tissues. According to this view, bioelectrical signals encode in their frequency pattern instructive rules that operate at various scales, ranging from individual cells to tissues and organs^1–6^. These signals have shapes resembling action potentials, but last much longer, and they involve an ensemble of synchronized cells. These electrical events last from a few seconds up to several minutes, thus rendering the term “spikes” inadequate for characterizing such electrical signals. The physiological role of bioelectrical signaling in non-electrogenic cells and tissues has been largely overlooked but is emerging as a new and promising field^7–9^as revealed by recent reviews about the critical role of ion channels and bioelectrical signaling in tissue regeneration, developmental biology^10–12^and cancer studies^13,14^.

To study bioelectrical signals used by non-excitable cells, a two-dimensional confluent monolayer of fibroblasts was selected. Fibroblasts have a prominent role in skin function and wound repair, a task that requires complex coordination of the cell population^9^. At the site of a wound, fibroblasts synthesize extra-cellular matrix proteins, influence the recruitment of immune cells, and differentiate into myofibroblasts to generate contractile forces that bring the wound edges together and promote wound closure^15–17^. It is known that dermal fibroblasts during wound healing coordinate cell activities and rely on the intricate interplay of ion channels, pores, and pumps, which generates a complex language of bioelectrical signals. In most cellular systems, it is accepted that the local calcium concentration, modulated by cell potential and voltage-gated ion channels, generates signaling waves. It has been postulated that tissues can form bioelectrical networks like neural networks, where the spatiotemporal distribution of membrane potentials is regulated by the state of individual cells and the intercellular connections, they establish^7^.

The relationship between bioelectricity and wounds goes back to the early days of electrophysiology in the 19th century, marked by pioneering experiments conducted by Galvani and Matteucci. When the skin was cut, a steady voltage gradient arose and persisted for hours at the wound edge, this voltage gradient was named the injury potential^18^. Injury potentials are direct current (DC) fields. In modern literature, they are also referred to as endogenous electric fields^19,20^.

It is accepted that electric fields play important roles in controlling several aspects of wound healing^21^. In this context, the extensive literature on the role of electrical signals in wound healing^22^tissue regeneration^23^ and the therapeutic potential of low-frequency electric fields on wound repair^24,25^ merits more in-depth investigation.

This study reports a bioelectronic-based method for monitoring dermal fibroblast activity during damage repair in a confluent cell population. Extracellular electrodes are used to measure electrophysiological signals from a confluent cell population. Our electrodes are custom-made large-area Multielectrode Arrays (MEAs) devices. Similar devices have been used to measure other non-excitable cell population activities such as astrocytes^26^ and C6 glia populations^27,28^. Our study aligns with recent findings showing that epithelial cells generate signals following a light-induced wound^29^. However, we provide an extended analysis and clear evidence of diverse bioelectrical signal patterns within dermal cell populations. Unlike the previous study on epithelial cells, we show that fibroblast populations not only produce signals in response to injury but also exhibit significant bioelectrical activity during monolayer formation. Furthermore, very distinct signal frequency patterns can be assigned to different cell activities, monolayer formation and wound repair.

Compared to existing methods such as optical fluorescence^30^our sensing platform has several advantages, including the ability to assess bioelectrical signals generated by cells in the ultra-low frequency region (10^− 4^ Hz), in real-time, non-invasively, and over periods longer than one week.

As bioelectrical signals may serve as instructive cues, it is reasonable to anticipate that populations of cells engaged in a specific task would exhibit distinct characteristics in their signal patterns. To investigate this premise, a population of fibroblasts was measured while engaged in two different activities. Initially, an unconnected cell population was seeded onto a sensing electrode, where they adhered and established a communicative fibroblast monolayer. Subsequently, in the ensuing phase, an intentional injury was induced by creating a controlled fissure within the confluent monolayer. The noticeable difference in the bioelectric frequencies observed in the corresponding patterns aligns with the view that bioelectrical oscillations integrate a cell language. The spectral properties embedded within bioelectrical patterns have the potential to lay the groundwork for unraveling the communication language employed by non-excitable cells, and how non-neuronal cells employ bioelectrical signals to convey information throughout tissues.

## Results

**Fig. 1:**
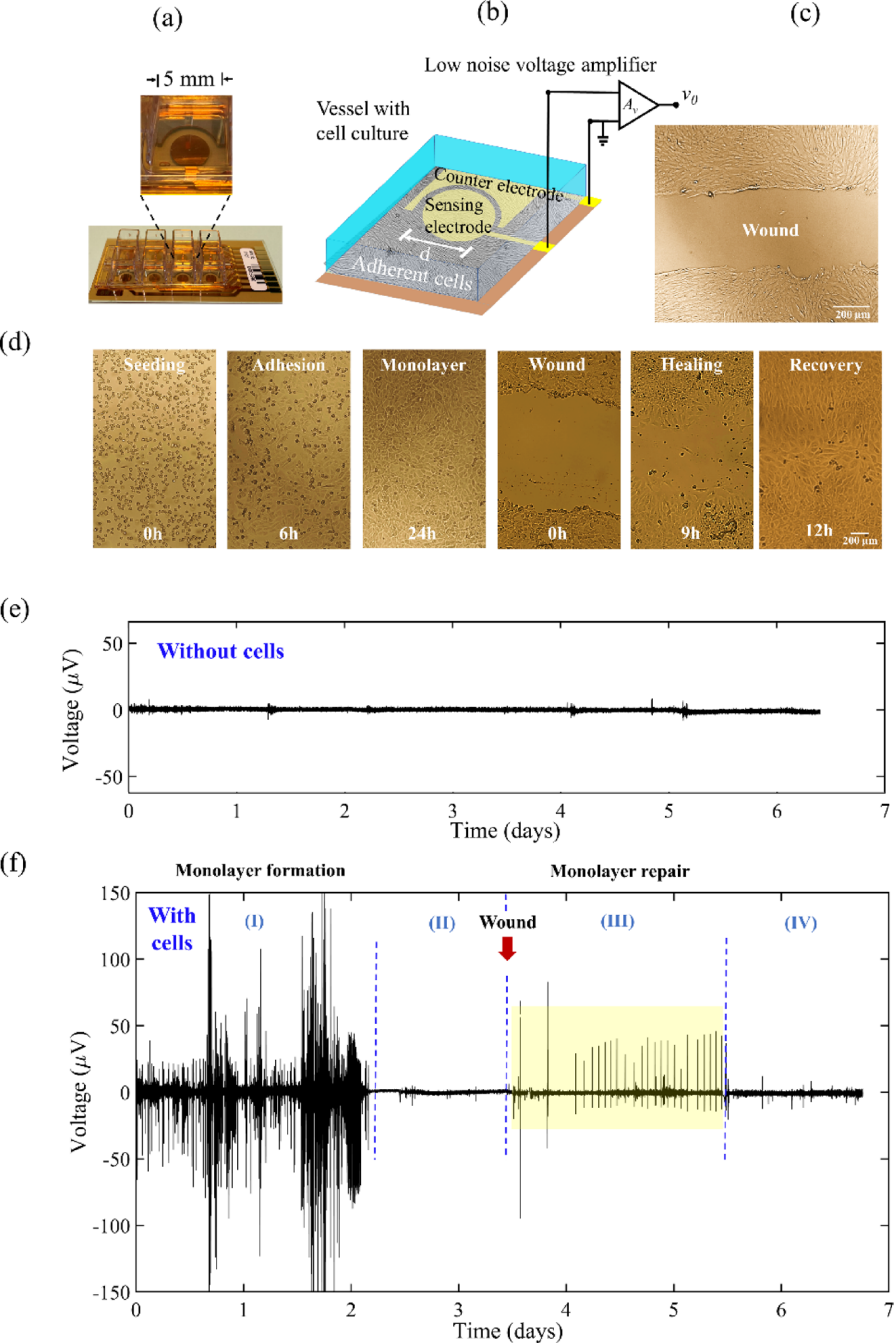
Sensing device and the sequential stages of cell monolayer formation, wound, and repair. (**a**) Photograph of the sensing device. (**b**) Schematic showing the device configuration and the electrical connections. (**c**) Photograph of a typical wound. (**d**) A series of images illustrating the sequential stages, starting from the formation of a cell monolayer, wound, the healing process, and ultimately, the recovery. (**e**) and (**f**) compare the electrophysiological time trace of an electrode without cells (baseline) with the activity of an electrode with an active cell population. In (f), the electrophysiological recording during monolayer formation extends over approximately the first two days (region I). This intense activity is followed by a quiescent region (region II). The wound is inflicted shortly after the third day, and the yellow-labelled quasiperiodic burst (region III) occurs a few hours after the injury. After this burst, which lasted almost two days, the cell population remained electrically quiescent (region IV).

The extracellular bioelectrical activity of a cell population, typically comprising between 3,000 and 4,000 cells, is measured by employing a planar electrode configuration and a low-noise electrophysiological recording system. A photograph of a sensing device and a schematic of the sensing electrodes, including their connections to the voltage amplifier are illustrated in Fig. 1 (a and b, respectively). The cells are arranged in a closely packed monolayer on top of the substrate, with a circular sensing electrode patterned alongside a counter electrode. A photograph of a typical wound made in a cell population monolayer is shown in Fig. 1c. During an electrophysiological experiment, continuous monitoring captures the various stages of the cell population, as illustrated in Fig. 1d. This set of photos depicts the progression from cell seeding through to the formation of a compact monolayer, the inflicted wound, the subsequent healing process, and finally complete recovery of the monolayer. The time for the formation of a monolayer is contingent upon the initial cell density, and the spatial width of the wound controls the temporal span required for complete wound recovery. In the case illustrated in Fig. 1d, the wound is closed 12 h post-injury. The representative images in Fig. 1c and d were captured for cells plated on plastic and not the gold surface of the measuring device, which is difficult to image.

The electrophysiological system relies on a low-noise voltage amplifier, where one of the inputs is grounded. The electrical stability of the ground connection is pivotal for maintaining a stable electrical reference point. Our measurement system has its own dedicated, low-impedance, and exceptionally stable ground terminal. The stability of our ground is corroborated by the electrophysiological baseline data displayed in Fig. 1e. Over a period spanning 6 days, we recorded a flat average noise level of approximately 4 µV peak-to-peak. We also underline that our amplifier is configured to operate in alternating current mode, filtering out any direct current drifts. Our electrophysiological system differs from electrochemical recording systems that utilize three-terminal electrodes, consisting of the counter, working, and reference electrodes. In electrochemical recording systems, the role of the reference electrode is to precisely determine the absolute potential between the working and counter electrodes. Additional information regarding the measurement methodology can be found in our previous study^31^.

### Overview of the bioelectrical signaling behavior from cell seeding to damage repair

Figure 1f provides an overview of the signalling behaviour exhibited by the cell population before and after the damage of a confluent monolayer. The time trace was recorded over 7 consecutive days. Initially, the time trace captures the spontaneous cell activity during adhesion and progression towards the formation of a fully confluent cell monolayer. This first phase is labelled as Region I and takes approximately 2.5 days. After initial intense activity associated with adhesion and spreading, the cell monolayer enters a relatively silent phase (Region II). 3.5 days after seeding the cells, the confluent cell monolayer was damaged using a soft plastic blade to create a fissure. The subsequent signalling activity during repair of the cell monolayer is indicated as Region III.

During the repair of the monolayer, a distinctive signal pattern emerges, exhibiting quasi-periodic behaviour with signals occurring at interval ranging from 60 to 100 min (0.27 to 0.15 mHz) (Region III). It is important to note that these quasi-periodic signal patterns are not initiated immediately after the damage but occur approximately around 4–6 h later. The ultra-low frequency quasi-periodic pattern persists for approximately 35 h. Subsequently, the cell population enters a quiet phase (Region IV) and visual inspection revealed that the damaged cell monolayer was fully repaired.

A summary of the overall cell signalling behaviour is as follows; cell bioelectrical activity is strong when cells are engaged in forming a monolayer. In the case of a healthy and stable monolayer, the bioelectrical activity is relatively weak. Both the processes of forming and repairing the monolayer involves biphasic signals. However, there are significant differences in the period of these signals in each situation. When forming a monolayer, the signals have a period below 3 min. The signals associated with monolayer repair have periods longer than one hour.

Six independent experiments were conducted to analyse the reproducibility of signals during monolayer formation. All signals within each 2.5-day-long electrophysiological time trace were used for data analysis (text S2, Figs. S2 and S3). Conversely, during the wound repair stage, only the region exhibiting ultra-low frequency patterns in the 6 experiments was considered for analysis (text S2, Fig. S4, S5 and S6). This decision was made because the remaining portions of the trace are silent or have sporadic signals, as illustrated in Fig. 1f.

The typical behaviour in most of our experiments involves a quasiperiodic pattern with an average frequency of 0.18 mHz. The pattern usually consists of signals with a strong upward component. In one experiment, however, we recorded a pattern of biphasic signals with identical frequency, but now with a strong downward component, as shown in the supplementary information on signal variability (Text S3, Fig. S7).

**Fig. 2:**
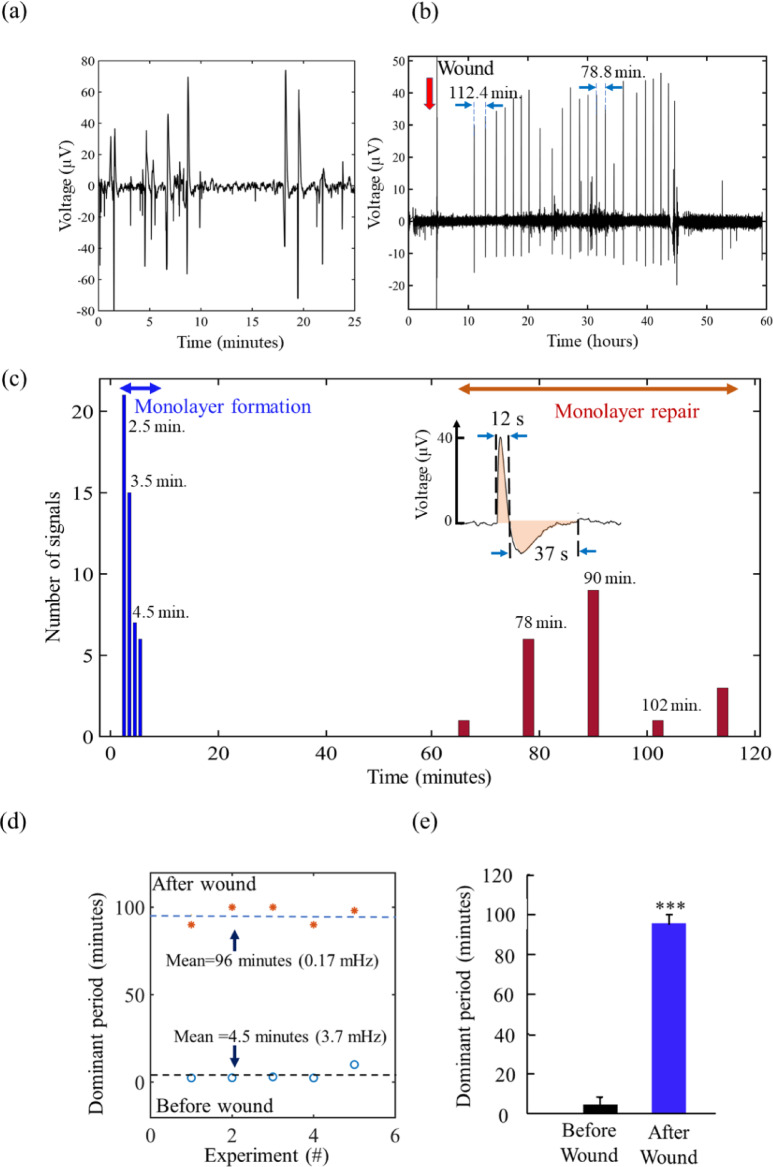
Comparison of the signal patterns during the formation of a monolayer and the subsequent repair process. (**a**) monolayer formation and (**b**) during repair. (**c**) A histogram of the period distribution of the signals. The bin width is 0.5 min (33 mHz) during the monolayer formation stage and 12 min (1.38 mHz) during the repair stage. The inset shows the shape of an individual signal. In (**d**), the dominant times associated with each biological activity across all six experiments are plotted. In this analysis, the bin width used varies between 1 and 2.5 min during monolayer formation and between 10 and 12 min during the repair stage. (**e**) the results of two side t-test performed for signal periodicity before and after wounding (*n* = 6 independent experiments) is represented. The difference between the means was highly significant (*p* < 0.001).

To quantify the spectral properties of signals during monolayer formation and the repair of the damaged monolayer, the collected data was analysed in the frequency domain. To perform the frequency domain analysis, the time interval between consecutive signals was measured and grouped into bins with appropriate temporal (or frequency) widths. The number of signals falling within each bin was then aggregated, and a histogram was constructed to represent the data. This procedure was adopted because the total number of signals during monolayer repair is relatively small (< 40 signals). A bin width of 0.5 min was used for the monolayer formation and a 12-minute bin width for the monolayer repair stage. Figure 2c displays a comparison of the signal period distributions obtained from measuring typical signals.

The relationship between cell function and signal behaviour can be described as follows. During the formation of the monolayer, the signal amplitude displayed high variability, and activity bursts were observed at frequencies within the range of a few minutes (approximately 2–3 min). Once the monolayer was formed and reached a stable state, the cells entered a relatively quiescent phase with reduced signal activity. However, during the repair process, a distinct and well-defined quasi-periodic signal pattern emerged. This pattern was characterized by a period spanning from 66 to 114 min and signals exhibited a consistent periodicity. Following injury, the signal pattern was comprised of a quasi-periodic activity consisting of distinct, well-defined signals that persisted for more than 30 h. All signals exhibited a nearly identical shape and had an approximate duration of 1 min. The shape of the signal consisted of a rapid and prominent upward component, lasting around 12 s, followed by a subsequent downward oscillation that gradually returned to the baseline over approximately 37 s. The correlation between a specific biological activity and a bioelectrical pattern relies on six independent experiments. Each of the experiments recorded the electrophysiological activity of dermal fibroblast cell populations for an extended duration of up to 6 consecutive days. For all the experiments, the spectral properties of signals were analysed following the histogram procedure described above and represented in Fig. 2c for one experiment. This analysis shows that a frequency prominently dominates, qualifying it as the dominant frequency. Apart from the histograms in Fig. 2c, six additional histograms are presented in text S2 (Fig. S2 and S3) for signal patterns during monolayer formation, and in text S2 (Fig. S4, S5 and S6a) for signal patterns after the wound are present. This behaviour was consistent during monolayer formation and monolayer repair. Consequently, two dominant signal periods (or frequencies), representing each of these biological processes, can reasonably be assigned to each experiment. To assess the consistency of the bioelectrical behaviour across all six experiments, the two dominant signal periods were plotted for each individual experiment (Fig. 2d and e). During the monolayer formation, the average signal period was 4.2 min with a standard deviation of 1.8 min During monolayer repair, the average period was 92 min, with a standard deviation of 7.9 min. The comparison of the average dominant period before and after the wound was performed using an independent t-test. The difference between the means was highly significant (*p* < 0.001).

### Details of the bioelectrical signaling during fibroblast monolayer formation

**Fig. 3:**
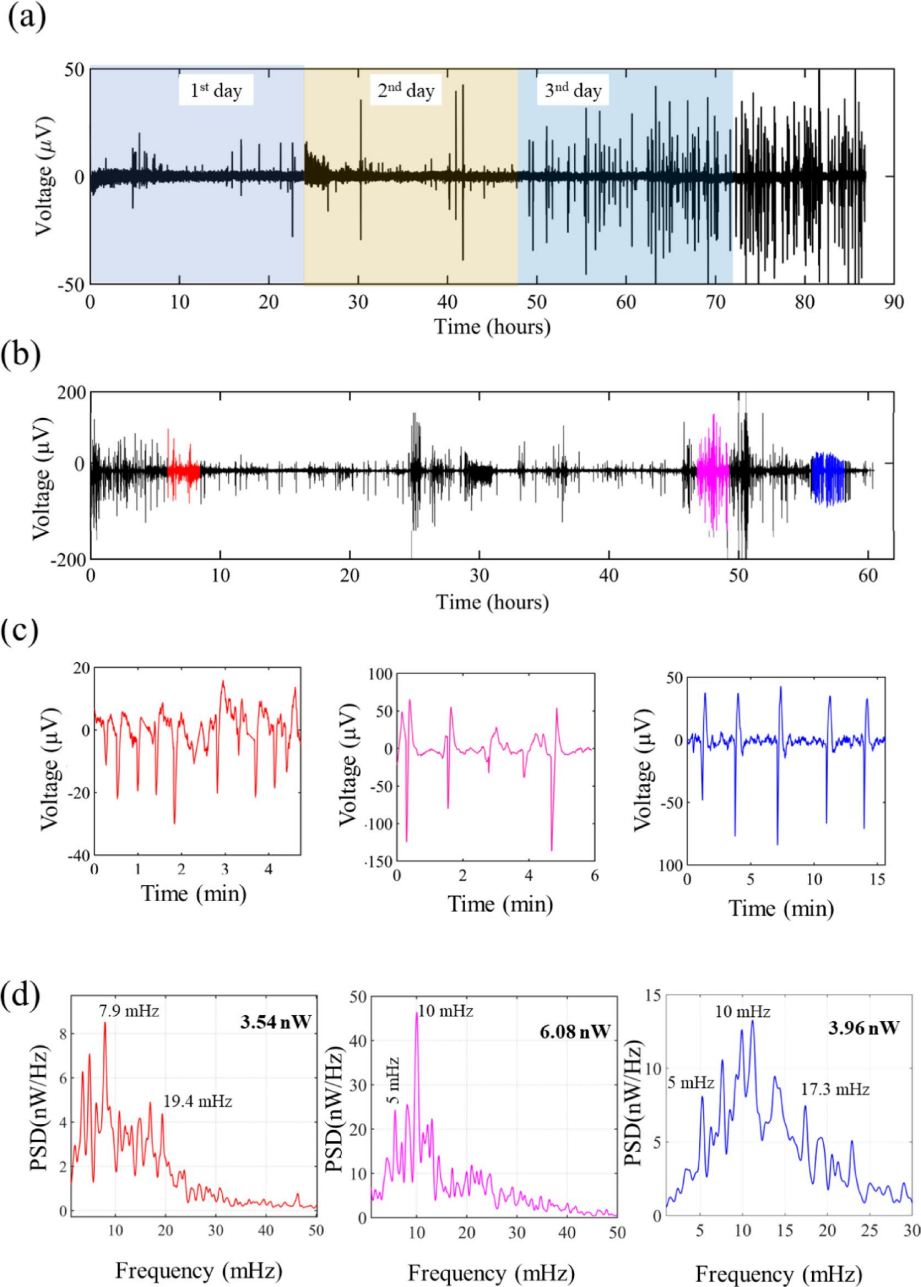
Bioelectrical patterns during the formation of a fibroblast cell monolayer. (**a**) Time trace of bioelectrical activity for a low cell density (< 300 cells/mm^2^). (**b**) Time trace for a high cell density (> 940 cells/mm^2^). (**c**) Detailed view of each selected region highlighted in different colors along the time trace in (**b**). (**d**) Power spectral density of the three selected regions. The electric power (in nano watts (nW)) of each spectrogram is indicated in the inset in bold.

In this section, a description of the bioelectrical signalling associated with monolayer formation is provided. The signal organization into patterns, typical frequencies, duration of patterns, and signal shapes are explored. Figure 3a and b describe in detail the bioelectrical activity during the formation of a compact monolayer. After seeding, signalling activity was not immediately initiated and the delay in activity was influenced by the initial cell density. The electrophysiological time trace in Fig. 3 shows the progression of the cellular response with time for different initial cell densities. Figure 3a for a low (< 300 cells/mm^2^) cell density and Fig. 3b for high (> 940 cells/mm^2^) initial cell density. Slower establishment of the compact monolayer permitted the signal evolution to be monitored in more detail. The trace in Fig. 3a consisted of discrete signals that gradually increased in amplitude before levelling off at ± 50 µV. Immediately after cell seeding, no signals were detected, and the first sporadic signals only appeared five hours after cell seeding. The first burst of activity was not observed until 24 h post-seeding and displayed quasi-periodic behaviour and signals were typically separated by quiet periods. At a higher cell seeding density, bioelectrical signals were detected significantly earlier (Fig. 3b). Despite the variability in cell seeding density, time zero in the electrophysiological records typically corresponds to one hour after cell seeding on the device.

To facilitate the interpretation of the electrophysiological time traces, three representative regions were identified (Fig. 3b) and are highlighted in different colours and a zoomed-in view (Fig. 3c) provides more details. The first region (red) consisted of monophasic downward signals, whereas the second region (magenta) displayed stronger signals with a biphasic shape. Signals in the third region (blue), exhibited a well-defined biphasic shape. The monophasic signals were relatively weak, with an average amplitude of 20 µV, while the biphasic signals reached amplitudes of up to 100 µV. The spectral analysis, (Fig. 3d), obtained by calculating the average power spectral density (PSD) with a frequency resolution of 0.5 mHz revealed the first burst of activity, comprised monophasic signals, with a dominant frequency of 7.9–8 mHz with satellite lines at 5.0 mHz and 19.4 mHz. In the middle region of the graph (magenta), the biphasic signals had a dominant frequency of 10 mHz. The strong biphasic signals in the last region of the graph also had a dominant frequency of 10 mHz. Additionally, the spectrum showed the presence of well-defined lines located at 5.0 mHz and 7.0 mHz alongside 17.3 mHz and 22 mHz bands. It is noteworthy that in the six replicas of the electrophysiological time traces that were recorded during monolayer formation (cell seeding density 625 cells/mm^2^, see text S2, Fig. S2 and S3) certain spectral lines aligned closely with the prominent 2.5-minute period (6.7 mHz) observed in the histograms in Fig. 2.

Overall, the spectral analysis demonstrated that, despite variations in signal shape and amplitude, the activity bursts were consistently characterized by dominant frequencies of 8 mHz and 10 mHz.

### Response to drug exposure

**Fig. 4:**
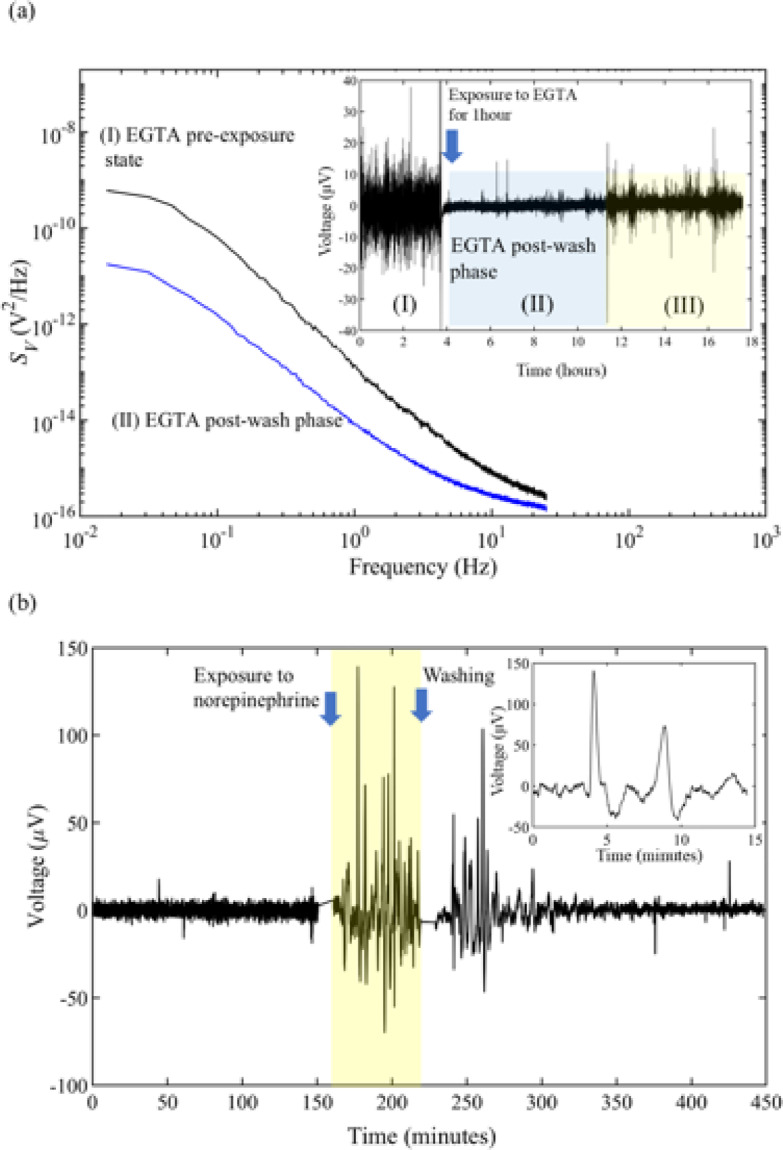
The bioelectrical response of dermal fibroblasts to EGTA and norepinephrine. (**a**) Compares the electrical activity before and after one hour of exposure to EGTA (10 micromoles per liter) in the frequency domain. The inset shows the EGTA effect in the time domain. (**b**) The electrical activity in response to norepinephrine (10 micromoles per liter), the time trace illustrates the activity before, during exposure, and after washing out. The inset is a detailed view of two discrete signals recorded during norepinephrine exposure.

To determine if drug exposure promotes a distinct bioelectrical activity of a confluent dermal fibroblast population two drugs were used; ethylene glycol-bis(β-aminoethyl ether)-,N, N’,N’-tetraacetic acid (EGTA), a calcium chelator that disrupts cell connections and norepinephrine, a hormone that according to previous reports interacts with skin fibroblasts^32,33^. Chemical stimulation was carried out on a confluent cell population with a bioelectrical activity characterised by weak random oscillations resembling noise but significantly higher than the intrinsic noise level of the sensing device. A segment of this bioelectrical phase is shown in the inset of Fig. 4a, region labelled (I). When the fibroblast population was exposed to 10 micromoles per liter of EGTA for 30 min, the bioelectrical activity decreased to less than half of its original value, as shown in segment (II) in the inset of Fig. 4a. The impact of EGTA represented in the frequency domain is illustrated in Fig. 4a. The noise power spectral density (PSD) decreased by about one order of magnitude for frequencies below 0.1 Hz. Only segment (II) was used in the PSD curve corresponding to the post wash out phase. Bioelectrical activity showed only partial recovery 8 h after EGTA wash out. The altered bioelectrical activity when EGTA was present in the culture medium confirms that Ca^2+^ and/or cell connectivity are essential for bioelectrical activity. This interpretation is supported by previous studies showing that extracellular EGTA can suppress intracellular calcium signaling. In NIH 3T3 fibroblasts, extracellular calcium chelation with EGTA abolished the intracellular transient calcium waves triggered by mechanical stimulation^34^. In HeLa cells EGTA blocks the second phase of histamine-induced calcium responses by inhibiting a non-IP3-dependent intracellular signaling pathway^35^. The slow recovery of the activity after EGTA treatment aligns with the time needed for cells to re-establish cell connections.

Figure 4b illustrates the bioelectrical response of a dermal fibroblast population to norepinephrine. Upon norepinephrine exposure (10 micromoles per liter), the noise level changed from an average of 12 µV to frequent, substantial discrete fluctuations surpassing 100 µV in amplitude, each signal lasting one to three minutes (inset of Fig. 4b). This behaviour persisted throughout the hour-long exposure period and continued for one hour after norepinephrine wash out, gradually diminishing over time.

## Discussion

To quantitatively determine the role of bioelectrical signals in the organization of monolayers of non-excitable cells, we carried out the characterization of signalling in dermal fibroblast populations. We used an in vitro wound-healing assay as a model system. Bioelectrical monitoring followed different stages of cell activity, starting from the formation of a cell monolayer to repair activity. Our analysis showed that each of these biological activities exhibited a distinct and unique bioelectrical pattern. Bioelectrical patterns associated with the monolayer formation predominantly exhibited frequencies in the range of 8 − 3 mHz (period between 2 and 5 min) while patterns associated with wound repair had a frequency of approximately 0.18 mHz (a period of 92 min). The two orders of magnitude separation between the two pattern frequencies allowed a clear assignment of each bioelectrical pattern to a specific biological activity.

The underlying biological significance of the signaling patterns has yet to be fully elucidated and there is still a long way to go before the reported bioelectrical patterns can be integrated into current biochemical theory. It is important to note that cells are exposed to a complex biomechanical environment during tissue repair, and it is plausible that mechanical forces activate specific ion channels. Supporting this, calcium imaging of human skin fibroblasts grown under two-dimensional conditions and subjected to mechanical stretch shows that the cells sense changes in hydrostatic pressure^36^. More specifically, members of the transient receptor potential (TRP) family of non-selective Ca^2+^ channels TRPC1, TRPM7, TRPV2 and TRPV4, which are known to be sensitive to stretch, were expressed in human skin fibroblasts and Synovial fibroblasts^37^. In view of this, the signaling patterns detected in the present study could be the result of downstream events resulting from specific processes (adhesion, migration, stretching, etc.) or alternatively be an instructional cue to coordinate activities across cell populations^8,38^.

Our electrophysiological-based devices capture several oscillating bioelectrical patterns. We sought to establish a correlation between the periods of the observed bioelectrical oscillations and other oscillatory behaviours, particularly those relevant to wound healing. The observation of spontaneous ultra low-frequency oscillations, named SELFOs has been identified in a variety of organisms spanning bacteria, hydra, and even human brains^22^. SELFOs are in the range of 0.01–0.1 Hz. Wound healing studies conducted both in vitro in epithelial cultures and in vivo in the epidermis of living mice revealed the presence of Erk waves^39–41^. These waves played a vital role in driving collective cell migration. However, it is noteworthy that the average speed of the Erk waves was approximately 2.12 μm/min., which does not align with the present data because the Erk wave would require approximately 16 h to cross the 2.1 mm diameter sensing electrode. Calcium waves reported in wounded fibroblast monolayers have average periods of 180 s (3 min), and signal durations from a few seconds up to 2 min^41–44^. These spectral properties match some bioelectrical signals recorded during monolayer formation but differ significantly from the patterns associated with monolayer repair. Other phenomena associated with cooperative cell migration are mechanical waves resulting from harmonized cellular motions. Epithelial cells confined in a 100 μm square (significantly smaller than our mm-scale sensor) exhibit mechanical waves with periods of 3 h. These disparities indicate that the bioelectrical oscillations detected during wound repair probably represent a distinct phenomenon. The frequency of some of the bioelectrical patterns identified were coherent with the time frame of biorhythms or clocks due to their long period and sustained presence over several consecutive days^45–47^. This is an aspect that deserves more research.

While the sensing electrodes capture signals from a substantial population of cells (> 3,000), the resulting electrophysiological time traces exhibit distinct and well-defined signals.

The data recorded allowed clear quantification of the time delay between a stimulus and the onset of a bioelectrical pattern. The 66–100 min bioelectrical pattern only began approximately 4–6 h after damage to the monolayer. Such time delays have been linked to the time required for the messages to travel, and the cells to prepare the machinery to accomplish the task. This reveals the relevance of electrophysiological devices for real-time monitoring of biological processes. The analysis of the signal amplitude in each bioelectrical pattern was also insightful, since the sensing electrodes measured a population of typically 3,000 cells, the discrete signals obtained implied the whole cell population or a subpopulation was synchronized. The entire cell population is expected to be involved in forming cell-cell connections as the confluent monolayer is established on the electrode. However, to repair a small, damaged region, only cells in the vicinity of the wound are in principle recruited. Cells located far away from the damaged region may not be actively enrolled in the repair. This view may explain why the signal amplitude during repair is lower than some of the signals detected during monolayer formation after cell seeding.

Our study is in line with the recent observation that epithelial cells generate signals after a light-induced wound^29^. However, our study provides evidence for the presence of diverse bioelectrical signal patterns within dermal cell populations. Cell populations do not only generate extracellular signals after a wound is inflicted but also are particularly active during the monolayer formation. Notably, the bioelectrical activities seemed to be part of a bioelectric lexicon, and signals were particularly pronounced when cell populations were actively engaged in specific tasks, while they appeared weak during periods of quiescence. The bioelectrical patterns exhibited a quasi-periodic nature within the ultra-low frequency spectrum [0.18 mHz 10 mHz]. Furthermore, the results describe how the collective behaviour of more than 3000 cells across several millimetres can be organized by patterns of electrical oscillations.

The electrophysiological-based method used in this study is non-invasive and can function over a cell population to provide a collective understanding of real-time bioelectric activities versus single cells. The advantages of the approach over classical fluorescence dyes were its high sensitivity, absence of toxic effects, and its use over extended time periods (weeks).

At present, it remains challenging to determine whether the bioelectrical signal patterns identified served as instructional cues or were the result of coordinated cell activity. If these patterns do indeed function as instructive signals, coordinating functions both within individual cells and across the monolayer, they may represent a fundamental property of tissues and biological organisms. This hypothesis aligns with the broader “bioelectric code” concept, which proposes that bioelectric signals play a crucial role in instructing tissue regeneration and development.

## Materials and methods

### Culture of human fibroblast cells

The immortalized fibroblast cell line, BJ-5Ta (ATCC CRL4001™) (Fig. 1c, S1), used in this study consisted of BJ foreskin fibroblast cells transfected with a pGRN145 hTERT-expressing plasmid. The cryopreserved cells were thawed, and cultures were initiated according to the supplier’s instructions. A 4:1 mixture of Dulbecco’s medium (Sigma-Aldrich, Germany) and Medium 199 (Sigma-Aldrich, Germany) supplemented with 10% fetal bovine serum (Sigma-Aldrich, Germany) and 0.01 mg/ml hygromycin B (Sigma-Aldrich, Germany) was used. Cells were maintained in a humid 5% CO_2_ incubator (Heraeus, Hanau, Germany) at 37 °C.

### Preparation of electrodes

For this study we used a modified 8W1E device (from Applied Biophysics, Inc company). The device was modified by removing the polymer on top of the electrode by incubating it with a 3% NaOH solution for 2 min, after which the electrode was washed with abundant water twice before being used. The typical electrode area used is 3.5 mm^2^. The size of the measuring electrode was confirmed by observation in a Leica DM IL microscope. Devices were exposed to ultraviolet light for 5 min prior to adding normal culture medium to the wells.

### Electrical measurements

The experimental electrical set-up had previously been optimized for 8WE1 devices^27^. The sensing device (8WE1) consisted of a patterned gold electrode on a polycarbonate (PET) substrate, that, for this study was adapted as described above and consisted of a chamber with a substrate area of 80 mm^2^ and a single circular electrode of 16 mm in diameter. The electrode surface area was large enough to lower the electrical double-layer resistance and the corresponding intrinsic thermal noise to levels typically below 3 µV. The underlying gold layout connected the active electrode area to contact areas outside the measuring chamber. For these experiments, the external interference was minimized by maintaining the sensor in a shielded metal box inside a humid 5% CO_2_ incubator (Heraeus, Hanau, Germany) at 37 °C and using low-noise cables. Extracellular voltage measurements were carried out using a low-noise voltage amplifier (SR 560, Stanford Research) with a gain set between 500 and 1000 and a dynamic signal analyzer (35670 A, Agilent). A detection window within the millihertz frequency range was used to measure signals with periods spanning from minutes to hours.

### Cell assays

For measuring fibroblast spontaneous bioelectrical activity, cells maintained in DMEM/M199 medium were seeded (625 cells/mm^2^) and allowed to settle for 15 min before connecting the device to the measuring system. In some control experiments, cell densities as low as 300 cells/mm² were used. Experiments were replicated in 6 independent assays, and the reproducibility of bioelectrical activity was assessed across several days during the formation of a confluent monolayer. For the wound healing experiment (*n* = 6), a small cross-shaped wound was created in the cell monolayer with the aid of a soft plastic blade, and detached cells were removed by washing three times in DMEM/M199 medium. New medium was added to the sensing device, and it was once again connected to the recording system, and the bioelectrical measurements were reinitiated. The medium was changed only after the wound was made. Although, if the experiment extended beyond three- or four days post-wounding, the medium was refreshed to sustain cell health. The evolution of wound healing was monitored by taking photos on a Leica DM IL microscope coupled to a Visicam PRO 20 C digital camera immediately and 48 h after the wound (Fig. S1c). To investigate alterations in bioelectrical activity following drug exposure, fibroblasts were challenged, with the extracellular addition of two drugs: ethylene glycol-bis(β-aminoethyl ether)-,N, N’,N’-tetraacetic acid (EGTA) with a concentration of 10 micromoles per liter for 1 h and norepinephrine (10 micromoles per liter) for 30 min. In both cases exposure was followed by a wash with normal medium. The background noise of the device and setup was measured using cell culture media, and the results were presented as time trace and power density spectrum (PSD).

### Statistical analysis

A two-sided t-test was performed to compare signal periodicity before and after wounding, using the Matlab function *ttest*.

## Supplementary Information

Below is the link to the electronic supplementary material.

Supplementary Material 1

## Data Availability

All data that support this study are available within the article and its Supplementary Information. Other relevant data are available from the corresponding authors upon request.
