# Supplementary material for: Extracellular bioelectrical lexicon: detecting rhythmic patterns within dermal fibroblast populations
Source: Sci Rep. 2025 Aug 14;15:29857. doi: 10.1038/s41598-025-15071-z (PMC12354770; doi:10.1038/s41598-025-15071-z)
Supplement: Supplementary file 1 — Supplementary Material 1 [file 41598_2025_15071_MOESM1_ESM.pdf]

Supplemental information (SI) for

Extracellular Bioelectrical Lexicon: Detecting Rhythmic Patterns within Dermal Fibroblast Populations

Rute C Félix *et al*.

*Corresponding author. Email: dpower@ualg.pt (D.M.P); hgomes@deec.uc.pt (H.L.G.)

This PDF file includes:

Supplementary Text S1 to S3

Figures S1 to S7

Supplemental Text S1

Details about confluent monolayers and the opening of fissures.

The immortalized fibroblast cell line, BJ-5Ta (ATCC CRL4001TM) are adherent cells that are organized in compact monolayers. The photographs in Figs. S1a) and S1b) highlight the closely packed nature of the cell monolayer, which contains elongated cells in close contact with their neighbours. A digital image of cells with a fluorescently labeled nuclei and fluorescently labelled cytoskeleton is shown in Fig. S1(b).

Supplemental Text S2

Details of ultra-low frequency patterns during monolayer formation and during wound repair.

In addition to the patterns highlighted in the main text, we present a detailed view of six ultra-low-frequency patterns (Figures S2 and S3) recorded during the monolayer formation stage. To determine the dominant frequency, we measure the interspike interval for each consecutive signal. Subsequently, these intervals are grouped into bins of predefined temporal width and represented as a histogram.

Four bioelectrical signal patterns recorded during the monolayer stage repair are presented in Figures S4 and S5. In certain electrophysiological time traces, pinpointing the duration of the ultra-low-frequency pattern poses a challenge, as illustrated in Figure S6, which presents a typical example. The histogram corresponding to the time trace in Figure S6 reveals a notable concentration of signals occurring at intervals ranging from 60 to 100 minutes, even when this pattern is not readily discernible through visual inspection. As elaborated in the manuscript, we speculate that these time traces may encompass two or more patterns generated simultaneously but operating out of phase.

Supplemental Text S3

Details about the signal variability.

The electrophysiological time traces exhibited variability. One source of variability is associated with the shape of the discrete signals in ultra-low-frequency signal patterns.

Figure S7 illustrates a case where the ultra-low frequency pattern matches the frequency documented in the main text but has a mirrored shape compared to those reported in the main text (Fig. 2c). Specifically, the high amplitude component of the signal is orientated downwards, whereas, in the signals mentioned in the main text, the high amplitude component is orientated upwards.

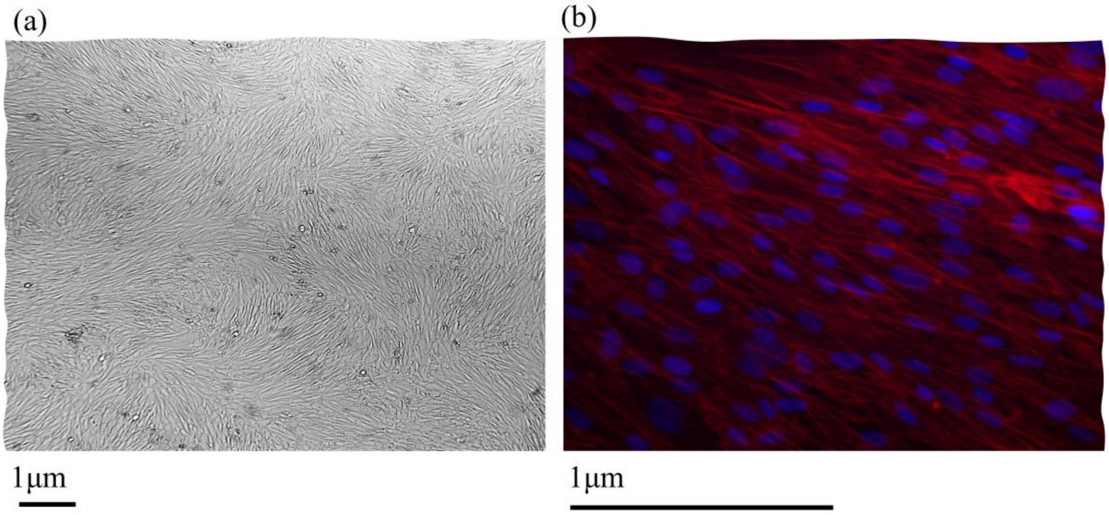

Figure S1. Optical photographs of a cell population. a) Typical compact monolayer. b) fluorescently labeled nuclei and cytoskeleton.

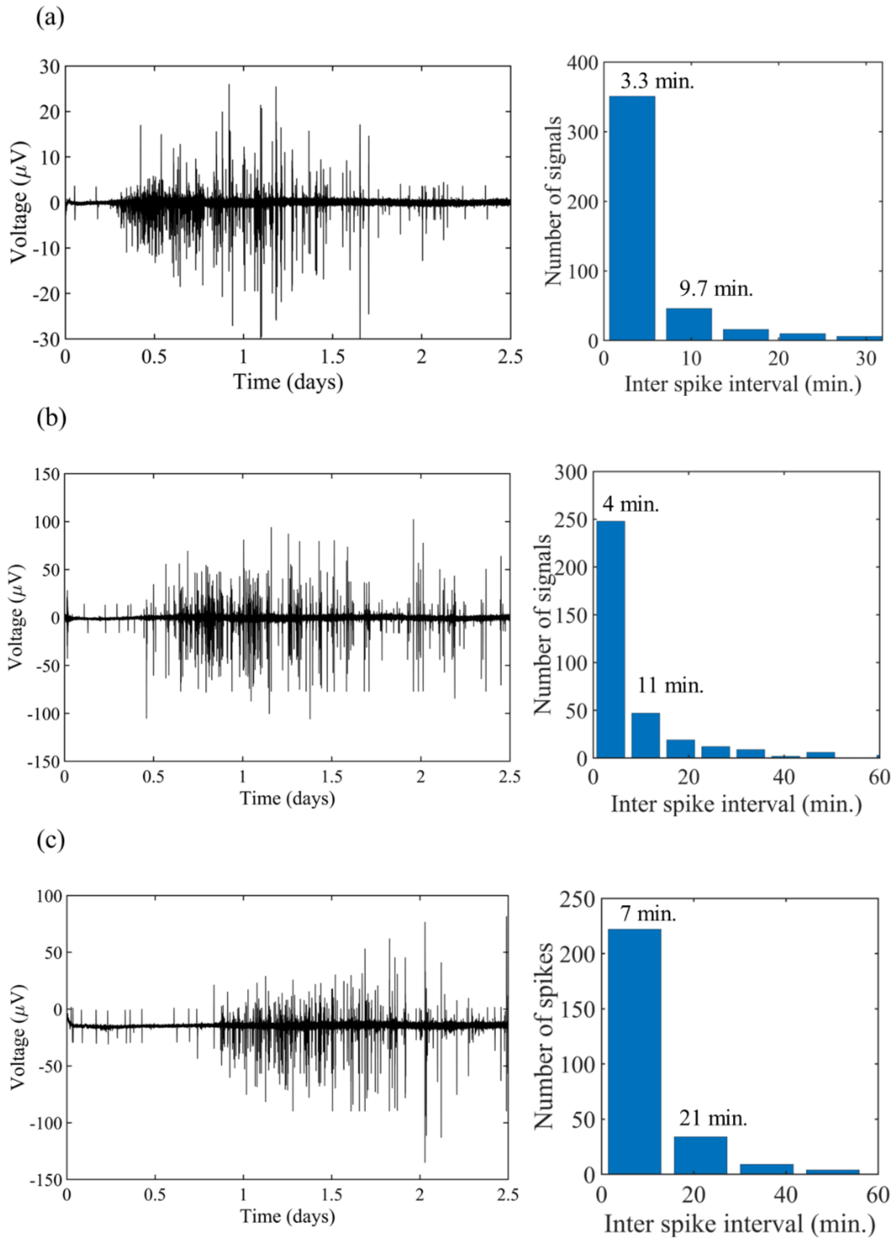

Figure S2. Bioelectrical signal patterns during monolayer formation and the corresponding distribution in slots of time are represented in the form of a histogram. In all the experiments the cell seeding density was kept constant (625 cells/mm2).

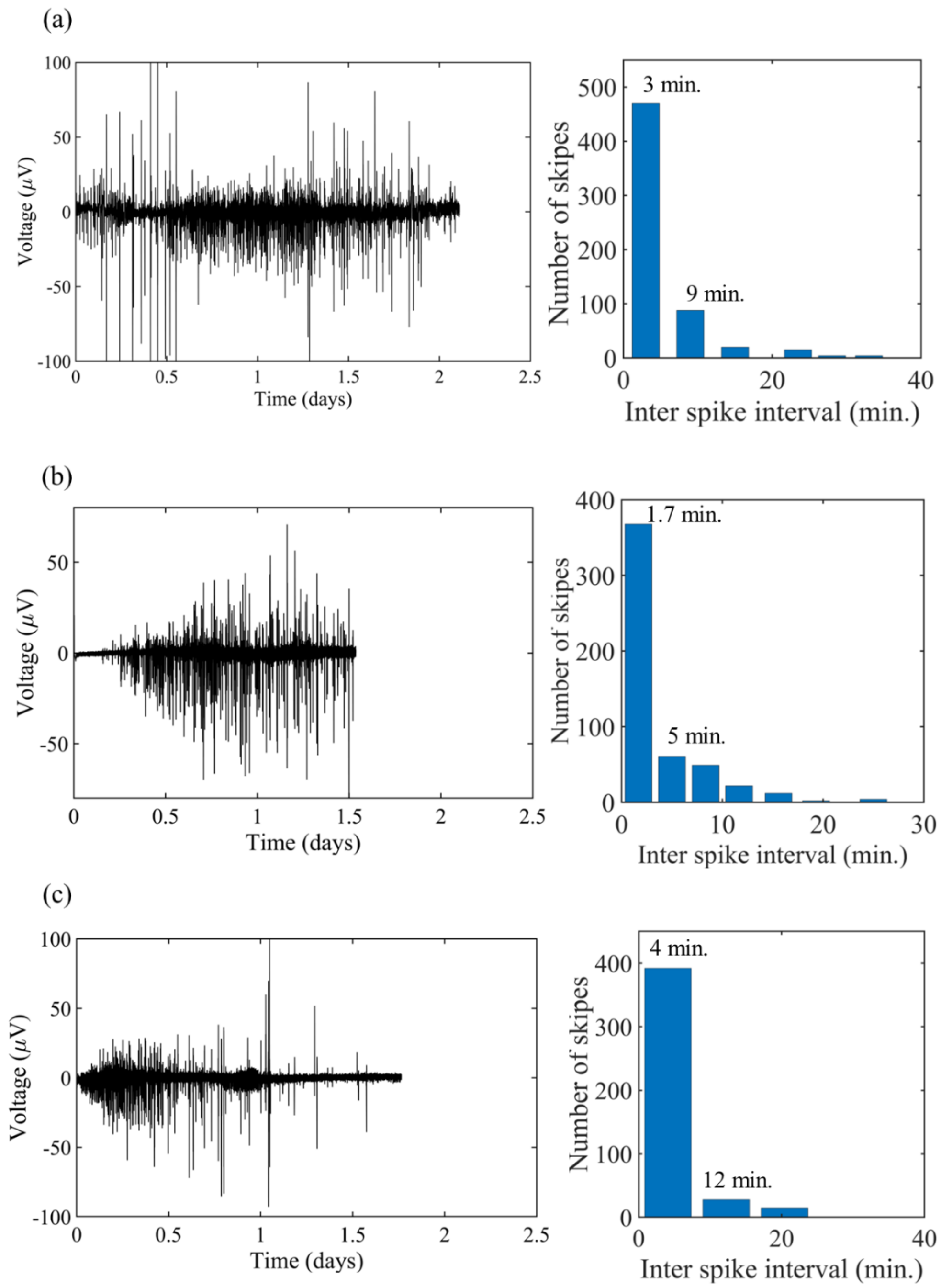

Figure S3. Bioelectrical signal patterns during monolayer formation and the corresponding distribution in slots of time are represented in the form of a histogram. In all the experiments the cell seeding density was kept constant (625 cells/mm^2)

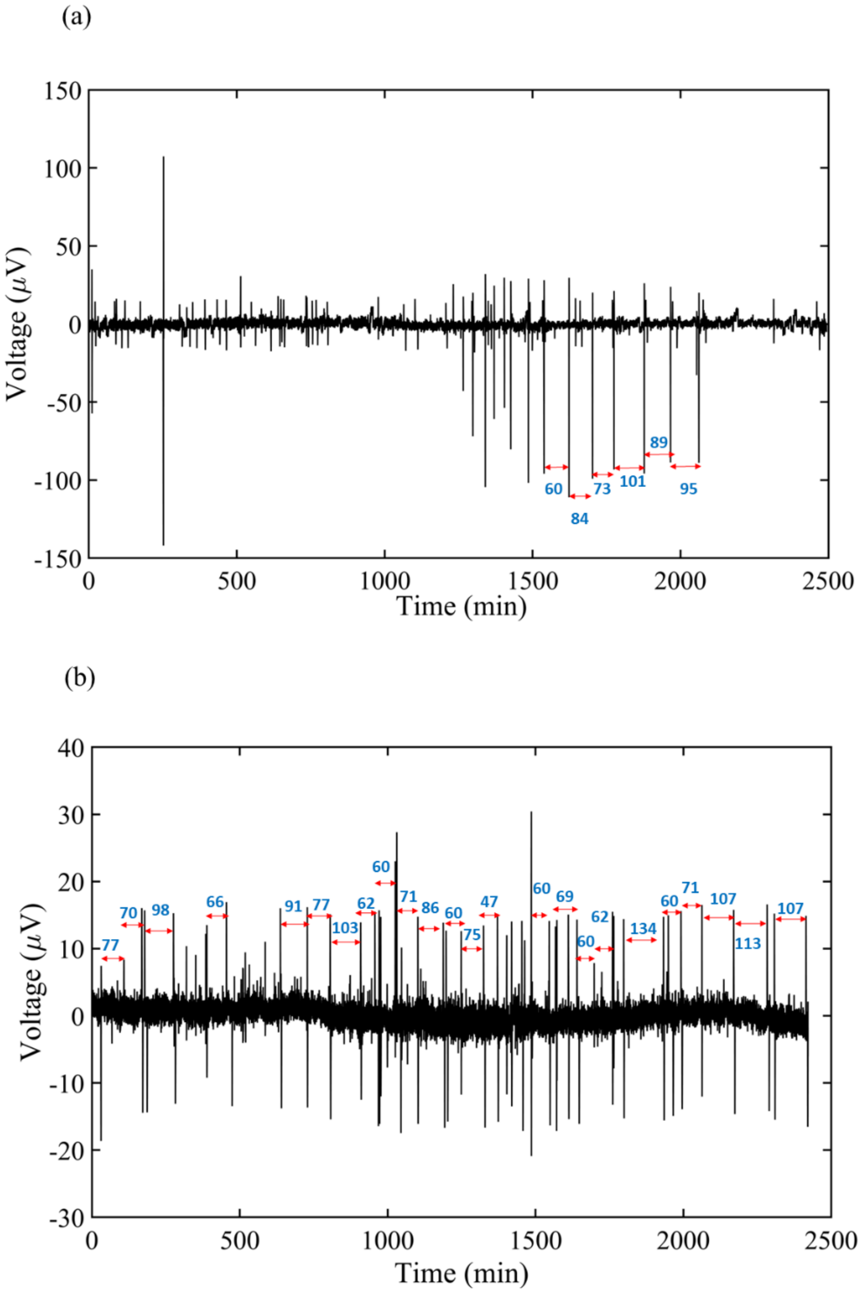

Figure S4. Detailed view of two ultra-low frequency patterns recorded after inflicting a wound. Time intervals between consecutive signals are marked. In all the experiments the cell seeding density was kept constant (625 cells/mm^2).

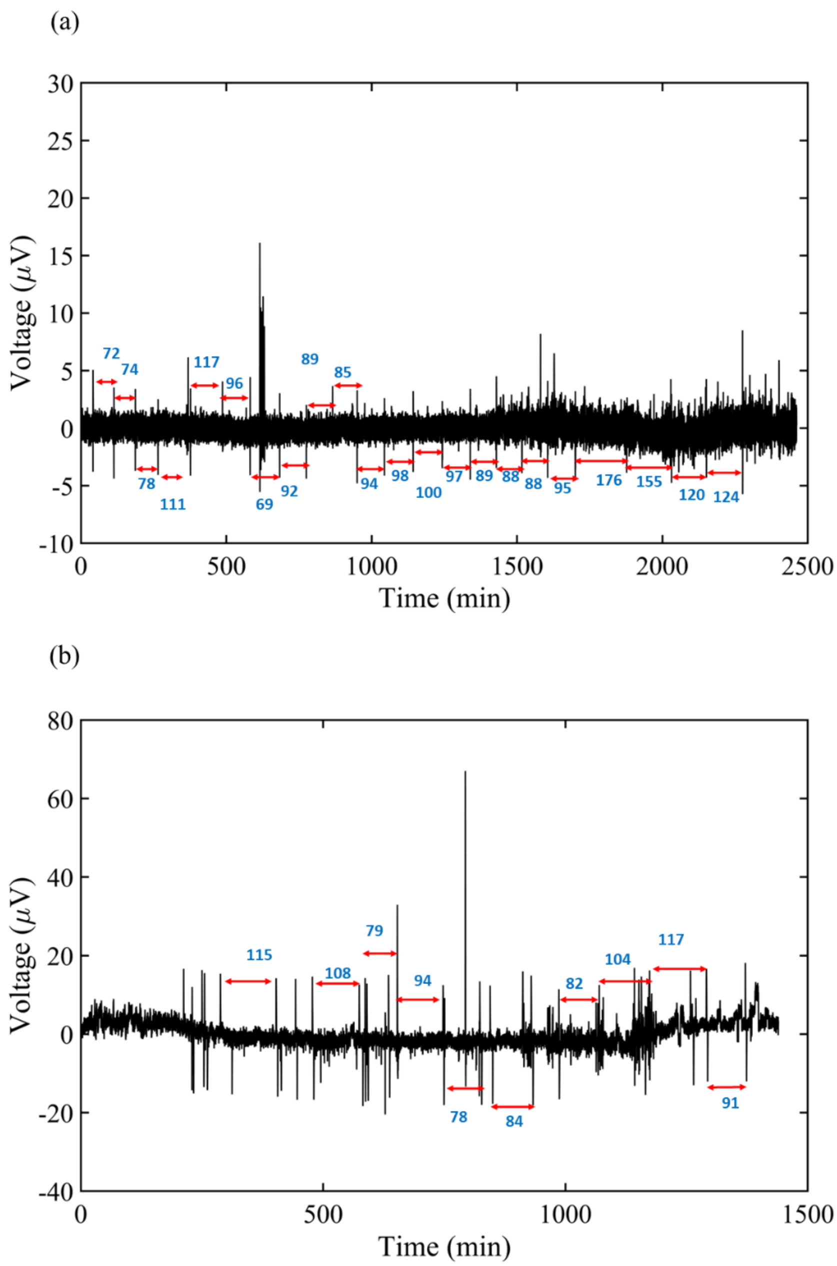

Figure S5. Detailed view of the ultra-low frequency patterns recorded after inflicting a wound. Time intervals between consecutive signals are marked. In all the experiments, the cell seeding density kept constant (625 cells/mm^2).

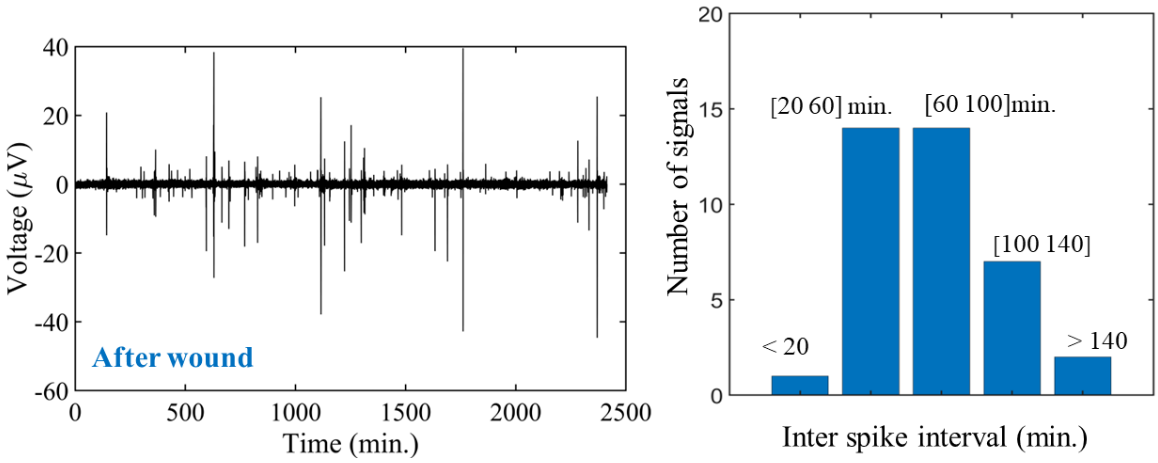

Figure S6. Detailed view of an ultra-low frequency pattern recorded after a wound was made and the corresponding representation of the interspike time interval in a histogram.

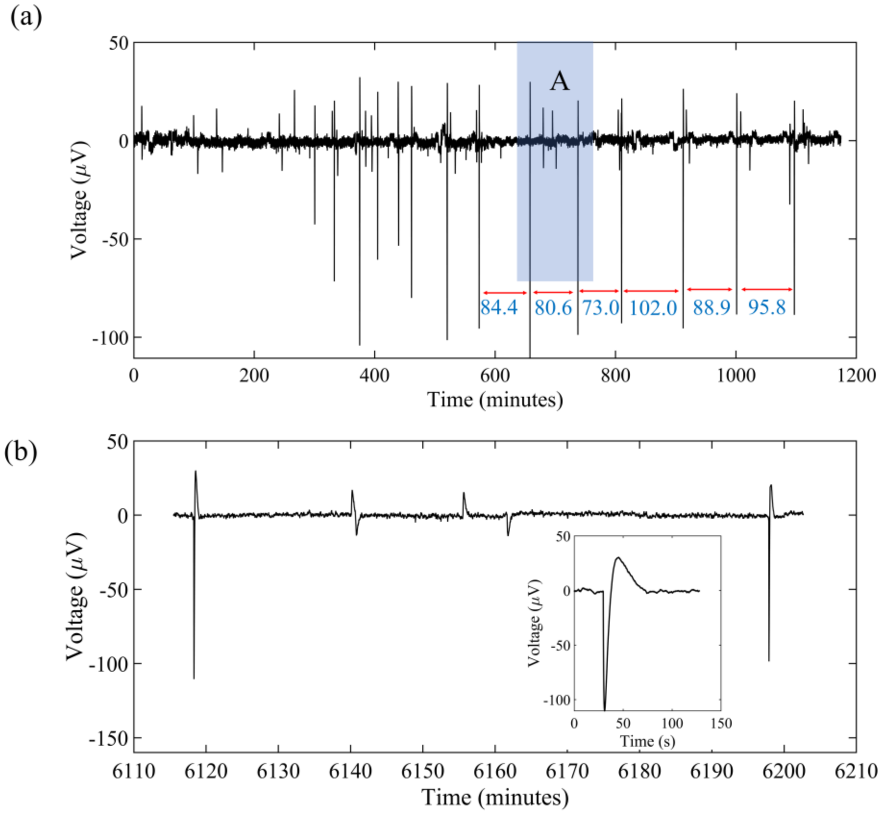

Figure S7. Variability in the shape of the ultra-slow burst recorded after creating a fissure in a confluent monolayer of dermal fibroblasts. a) the average interval between signals is 87 minutes (0.19 mHz). (b) is a zoom view of region A highlighted in (a). The inset in (b) depicts the shape of an individual signal. This signal shape is a mirror image of the signals in Figure 1f of the main article.
